# Hippocampal Interaction With Area 25, but not Area 32, Regulates Marmoset Approach–Avoidance Behavior

**DOI:** 10.1093/cercor/bhz015

**Published:** 2019-02-23

**Authors:** Chloe U Wallis, Gemma J Cockcroft, Rudolf N Cardinal, Angela C Roberts, Hannah F Clarke

**Affiliations:** 1 Department of Physiology, Development and Neuroscience, University of Cambridge, Downing Street, Cambridge, CB2 3DY, UK; 2 Department of Psychiatry, Box 189, Level E4, Cambridge Biomedical Campus, Cambridge, CB2 OQQ, UK; 3 Liaison Psychiatry Service, Cambridge and Peterborough NHS Foundation Trust, Box 190, Cambridge Biomedical Campus, Cambridge, CB2 OQQ, UK

**Keywords:** anterior hippocampus, decision making, marmoset, medial prefrontal, subgenual cingulate

## Abstract

Affective disorders are associated with increased sensitivity to negative feedback that influences approach–avoidance decision making. Although neuroimaging studies of these disorders reveal dysregulation in primate cingulate areas 25 and 32 and the anterior hippocampus (aHipp), the causal involvement of these structures and their interaction in the primate brain is unknown. We therefore investigated the effects of localized pharmacological manipulations of areas 25 and 32 and/or the aHipp of the marmoset monkey on performance of an anxiolytic-sensitive instrumental decision-making task in which an approach–avoidance conflict is created by pairing a response with reward and punishment. During control infusions animals avoided punishment, but this bias was reduced by increasing glutamate release within the aHipp or area 32, and inactivation or 5-HT1a antagonism within area 25. Conversely, increasing glutamate release in area 25 enhanced punishment avoidance but, in contrast to previous reports, area 32 and aHipp inactivations had no effect. Simultaneous inactivation or 5-HT1a antagonism within area 25, but not area 32, abolished the reduced punishment avoidance seen after increasing aHipp glutamate. Besides providing causal evidence that these primate areas differentially regulate negative feedback sensitivity, this study links the decision-making deficits in affective disorders to aberrant aHipp–area 25 circuit activity.

Depression and anxiety are affective disorders that change emotions and decision making to profoundly affect quality of life. The decision-making changes are seen in the context of both positive and negative emotion, and manifest as increased avoidance of threats or punishments, and decreased approach to potential rewards (Dickson and MacLeod 2004; Dickson 2006). Experimental studies of the neurobiology and neuropharmacology of affective decision making in rodents, primates and humans have used approach–avoidance paradigms, in which rewards and punishments are pitted against each other to jointly influence decisions (Amemori and Graybiel 2012; Bach et al. 2014; Clarke et al. 2015; Ito and Lee 2016). Neurally, these tasks have implicated several regions of the prefrontal cortex (PFC) including the orbitofrontal cortex (OFC) and ventrolateral PFC (vlPFC) and particularly the anterior/ventral hippocampus (a/vHipp) in approach–avoidance behavior in humans, rats and monkeys humans/rats (Aupperle and Paulus 2010; Clarke et al. 2015; O’Neil et al. 2015; Ito and Lee 2016; Schumacher et al. 2016).

There is also growing interest in the monosynaptic, glutamatergic circuit that links the aHipp to the medial PFC (mPFC) in rats, monkeys, and humans (Godsil et al. 2013)—in particular, Brodmann’s anterior cingulate areas 25 and 32, which show changes in morphology and activity that are prominently associated with depression and anxiety disorders (Ito et al. 1996; Mayberg et al. 2000; Greicius et al. 2007; Hamani et al. 2011; Fonzo et al. 2014). While the aHipp is independently implicated in both approach–avoidance decision making and the symptoms and treatment of depression and anxiety (Tsetsenis et al. 2007; Aupperle and Paulus 2010; Godsil et al. 2013; O’Neil et al. 2015), mounting correlative evidence suggests that interactions between the aHipp and mPFC are crucial for negative emotion regulation. In depressed humans, increased aHipp activation predicts increased activity in area 25 (Hamilton et al. 2011), and connectivity changes in an aHipp–mPFC circuit (including area 25) are implicated in the recovery from post-traumatic stress disorder and anxiety disorders (Dickie et al. 2011). Stress—a known trigger for anxiety and depression—also decreases the functional coupling between the aHipp and the mPFC, and is associated with reduced volume of the aHipp and the subgenual cingulate cortex (Admon et al. 2013; Treadway et al. 2015). Such coupling is strongly dependent upon serotonin (5-hydroxytryptamine, 5-HT) integrity (Puig and Gener 2015) and patients who respond to serotonergic antidepressants show differences in the strength of coupling between the aHipp and area 25 compared with nonresponders (Seminowicz et al. 2004). This process may be mediated by 5-HT1a receptors, which are densest in area 25 (Palomero-Gallagher et al. 2009), and are known to modulate negative emotion in both area 25 and the hippocampus (Tsetsenis et al. 2007; Albert et al. 2014; Ramirez-Mahaluf et al. 2015).

Despite this strong, correlative evidence for the importance of an aHipp–area 25 circuit, it has not been causally investigated in a primate brain. Studies in rodents have highlighted the importance of aHipp–mPFC connectivity in the control of anxiety related behavior (Adhikari et al. 2010; Padilla-Coreano et al. 2016) but they have either not differentiated between cytoarchectonically distinct regions within the mPFC (i.e., prelimbic [PL] and infralimbic [IL]) or have selectively targeted the more dorsal PL region (Sierra-Mercado et al. 2010; Adhikari et al. 2010, 2011; Sotres-Bayon et al. 2012; Padilla-Coreano et al. 2016). However, based on evidence from both anatomical connectivity and cytoarchitectural studies, PL shows homology with 32, and IL with 25 (Vogt and Paxinos 2014; Heilbronner et al. 2016), and thus while the human studies emphasize the importance of aHipp–area 25 (IL) circuitry, rodent studies appear primarily to highlight aHipp–PL (area 32) circuitry. On the other hand, areas 25 and 32 are clearly delineated in the primate brain and recent behavioral findings in marmoset monkeys showed that inactivation of areas 25 and 32 had the opposite effect in fear discrimination and extinction to their putative rodent homologs, indicating uncertainty over the functional analogy between IL/25 and PL/32 (Sierra-Mercado et al. 2010; Wallis et al. 2017). Given that decision making deficits are core symptoms in anxiety and depression, understanding how aHipp connectivity with mPFC areas 25 and 32 contributes to approach–avoidance behavior in primates is therefore crucial to both understanding, and alleviating the burden of these debilitating and costly disorders.

To address these questions, we determined the effects of temporarily manipulating the aHipp and the aHipp–mPFC circuitry of the marmoset monkey (by simultaneously manipulating areas 25 or 32 and the aHipp) on an anxiolytic-sensitive approach–avoidance behavioral paradigm developed specifically for non-human primates (Clarke et al. 2015) (Fig. 1). As human studies indicate that both overactivation (Hamilton et al. 2011) and sclerosis (Bach et al. 2014) of the aHipp, and altered 5-HT1a-mediated transmission are associated with decision-making abnormalities, we used anatomically specific intracerebral infusions of drugs designed to cause neuronal inactivation (GABA agonists that inactivate cell bodies), activation (presynaptic disinhibition of glutamate release), or 5-HT1a antagonism to investigate their contribution to decision making. For control purposes, and given the importance of areas 25 and 32 for the regulation of emotion, we also determined the effects of manipulating areas 25 and 32 independently.

**Figure 1.**
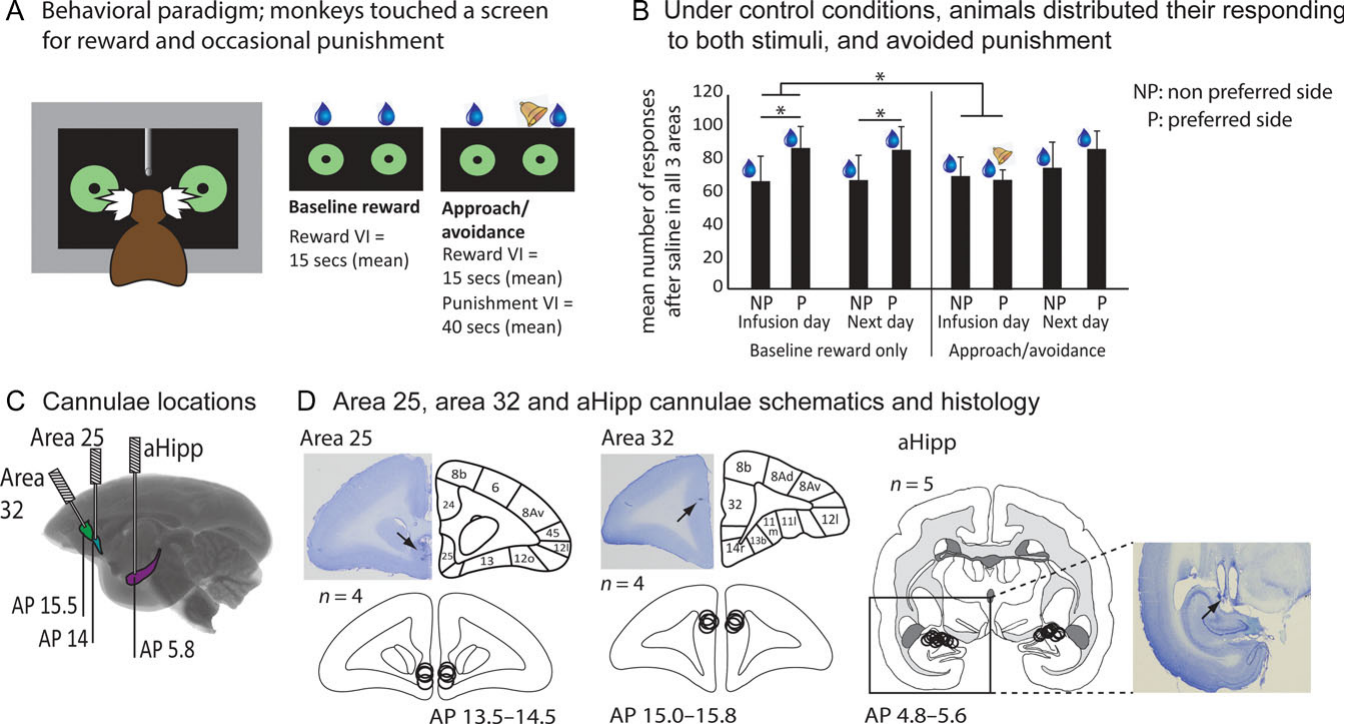
Approach–avoidance paradigm and prefrontal cannulae placements. (*A*) Marmosets were presented with two identical visual stimuli presented to the left and right of a touch-sensitive computer screen. Touching either stimulus earned reward (5 s banana milkshake; blue drop) according to independent but identical reward schedules. Occasionally and in addition to the reward schedule, responding to one of the stimuli also earned a punishment (0.3 s 117 dB mildly aversive loud noise; bell) on a leaner independent variable interval schedule. (*B*) Under control conditions marmosets responded relatively equally to both stimuli, with a slight preference to their preferred side (P). Accordingly there is more responding to the preferred/to-be-punished side than the nonpreferred (NP)/nonpunished side under baseline conditions that is seen on both the infusion day and the day after (infusion day, *t*_4_ = 3.5, *P* = 0.024; next day, *t*_4_ = 3.03, *P* = 0.038). This bias was abolished when punishment was introduced on the preferred side as animals biased away from the punishment, towards the nonpreferred/nonpunished side. However, the day after the punishment/saline infusion, the preferential responding to the preferred side returned, and did not differ from equivalent baseline performance (F_1,4_ = 2.58, *P* = 0.18). Data represent mean number of responses after saline infusion into the aHipp and areas 25 and 32 ± standard error of the mean (SEM) (*n* = 5). *P < 0.05. (*C*) Sagittal marmoset MRI section illustrating the rostrocaudal targets of area 25, area 32, and aHipp infusions. (*D*) Schematics showing the intracerebral cannulae targeting area 25, area 32, and the aHipp (in the PFC areas, the double cannulae straddled the midline resulting in each unilateral area receiving a single cannula), the actual cannulae locations for each animal and representative histological sections with arrows marking the position of the cannulae. All cannulae are plotted onto a single coronal section for each target area and the AP range indicated. Cytoarchitectonic parcellation is according to Burman and Rosa (2009) and the circles represent the estimated maximal spread of the muscimol/baclofen or saline infusions (West et al. 2011).

## Materials and Methods

### Subjects and Housing

Five experimentally naïve marmosets (*Callithrix jacchus*; *n* = 2 females, *n* = 3 males), bred on site at the University of Cambridge Marmoset Breeding Colony, were housed in male/female pairs (males were vasectomized). They were kept in a 12-h light-dark cycle (lights on at 7:00 AM, lights off at 7:00 PM) in a controlled environment of 22 ± 1 °C in temperature and 50 ± 1% humidity. Their cages contained a variety of environmental enrichment aids including suspended ladders, ropes and wooden branches to climb and swing on, and boxes to play in. All monkeys were fed 20 g of MP.E1 primate diet (Special Diet Services) and 2 pieces of carrot 5 days a week after the daily behavioral testing session, with simultaneous free access to water for 2 h. On weekends, their diet was supplemented with fruit, rusk, malt loaf, eggs, bread, and treats, and they had free access to water. All procedures were carried out in accordance with the UK Animals (Scientific Procedures) Act 1986 and the University of Cambridge animal welfare and ethical review board under project license PPL 70/7618.

### Approach–Avoidance Paradigm

Behavioral testing took place within a sound-attenuated box in a dark room as described previously (Clarke et al. 2015). Animals were trained to enter a transparent Perspex carry box for marshmallow reward, in which they were transported to the behavioral test apparatus. The Perspex carry box was placed inside the test chamber, and one side of the box was removed to allow the marmoset to reach through an array of vertical metal bars to touch stimuli presented on a touch-sensitive computer screen (Campden Instruments). The test chamber was lit by a 3-W house light located in the ceiling of the chamber and contained a computer-controlled siren generator (120 dB; Biotronix) through which a brief (0.3–0.6 ms) siren could be played, and a computer-controlled central spout through which a reward (5 s) of cool banana milkshake (Nestlé) could be delivered. The apparatus was controlled by the Whisker control system (Cardinal and Aitken 2010) and in-house software (K. Braesicke and R. Cardinal). Three video cameras were positioned in the test chamber so that the marmoset could be observed by the experimenter during testing.

After acclimatization to the testing apparatus, marmosets were familiarized with the milkshake reward and taught to respond to the touchscreen by placing marshmallow on the screen as described previously (Roberts et al. 1988). Once they were reliably making 30 or more accurate responses to a green square presented on either side of the screen, the stimuli were changed to green circles. A variable interval (VI) schedule was then introduced gradually for each stimulus independently, until the monkeys were responding to both stimuli approximately equally on a mean schedule of 15 s (ranging from 10 to 20 s in increments of 5 s). This means that, on average, a reward was available on each independent stimulus every 15 s (the VI delay), and the first response made after that interval has elapsed results in the delivery of reward. If responses were made before the interval had elapsed they were recorded but did not elicit reward (unrewarded responses) and had no impact on the underlying VI schedule. On such VI schedules when the timing of the reward is unpredictable and there is a relatively weak relationship between absolute levels of responding and reward, animals typically show a steady state of responding on both stimuli across the session to optimize how much reward they obtain. This schedule therefore involves only approach responses and is the baseline reward condition.

To introduce the avoidance component, we then introduced a punishment on one side only. This punishment was a brief loud noise known to be mildly aversive to marmosets (Mikheenko et al. 2015). During training, initial presentations of this loud noise were at 90 dB, but incremented slowly up to 117 dB with little or no effects on the number of responses performed. Ultimately, the punishment was presented on one side only, on a mean schedule of 40 s (ranging from 20 to 60 s in 5 s increments; less frequent than the baseline reward schedule), and was superimposed on top of the usual baseline reward schedule. This is the approach–avoidance condition. In this condition a response to one side could produce punishment, reward or nothing, and a response to the other side could only produce reward, or nothing. Thus for a given response to a particular stimulus, the two independent VI schedules for reward and punishment ensure that the animal does not know what it is going to get, or when it is going to get it. This therefore sets up a classic approach–avoidance conflict, as after experiencing the punishment on a particular side they have a choice. They can either continue approaching the punished side and therefore receive both the punishments and the associated rewards, or avoid the punished side and lose out on those rewards. The use of a VI schedule on the nonpunished side ensures that they cannot simply compensate for this loss by responding more on the nonpunished side. It should be noted that if both the VI schedule for punishment and reward had timed out before the animal’s next response in the approach–avoidance condition, then the animal received the outcome from whichever schedule had timed out first. Another response was then required before obtaining the outcome from the other schedule. This ensured the animal did not receive punishment and reward simultaneously.

The animals received the baseline reward condition on most days, and the approach–avoidance condition no more than twice a week. As the monkeys are always waiting for the next reward on each side, they distribute their responding approximately equally to both sides of the screen on the baseline reward schedule, with only minor intrinsic preferences (biases) to one of the sides (see Supplementary Fig. S1b). However, to avoid any intrinsic spatial bias contributing to a punishment-induced bias, the punishment was always introduced onto the marmosets’ “preferred side” (the side on which they had made the most responses the day before; see Fig. 1*B*). If a response was rewarded, the stimulus remained on the screen for the duration of the reward (5 s). If a response was unrewarded, the stimuli disappeared and then immediately reappeared without altering the underlying VI schedules. If a response was punished, the stimuli disappeared, the aversive noise sounded (0.3–0.5 s), and the stimuli immediately reappeared as before. The session length was 12 min and each animal was tested once per day, 5 days a week. Once the monkeys were all making a consistent number of responses on the task every day and responding relatively equally to both sides of the screen they were considered “trained,” and underwent cannulation surgery.

### Cannulation Surgery

Marmosets were premedicated with ketamine hydrochloride (Vetalar; 0.05 mL of a 100 mg solution, i.m.; Amersham Biosciences and Upjohn, Crawley, UK) before being given a long lasting nonsteroidal, anti-inflammatory analgesic (Carprieve; 0.03 mL of 50 mg/mL carprofen, s.c; Pfizer, Kent, UK). They were intubated and maintained on 2.0–2.5% isoflurane in 0.3 L/min O_2_ and placed into a stereotaxic frame modified for the marmoset (David Kopf, Tujanga, CA). Pulse-rate, O_2_ saturation, breathing rate, and CO_2_ saturation were all monitored by pulse-oximetry and capnography (Microcap Handheld Capnograph, Oridion Capnography Inc., MA, USA), and core body temperature was monitored by a rectal thermometer (TES-1319 K-type digital thermometer, TES Electrical Electronic Corp., Taipei, Taiwan). Cannulae (Plastics One) were implanted into area 25 (double 7-mm-long cannulae, 1 mm apart, anteroposterior [AP] + 14, lateromedial [LM] ± 0.5), area 32 (double 2 mm long cannulae, 1 mm apart; AP + 17; LM ± 0.5 at a 30° AP angle), and the aHipp (double 15-mm-long cannula in each aHipp, 1 mm apart, AP + 6, LM ± 5.75/7.75, ventral + 5). When fully recovered postoperatively, all monkeys were returned to their home cage and then received the analgesic meloxicam (0.1 mL of a 1.5 mg/mL oral suspension; Boehringer Ingelheim) for 3 days as well as 10 days of “weekend diet” and water ad libitum to ensure complete recovery before returning to testing. Cannulae were cleaned with 70% ethanol during every infusion and at least once every week (and caps and cannula blockers changed) to ensure the cannula site remained free from infection. Figure 1*C*,*D* illustrates the cannulae targets. After euthanasia and histological assessment (Wallis et al. 2017), one animal was found to be cannulated in the septum instead of area 25, and another animal was only cannulated unilaterally in area 32. Their data for these areas are excluded. There was no evidence that the infusions caused cell death in any of the target areas.

### Drug Treatments and Drugs

Approximately twice a week before behavioral testing, animals received infusions of drugs or vehicle down the cannulae in order to determine the contribution of aHipp–mPFC circuitry and 5-HT1a receptors to approach–avoidance decision making. In this way, the use of chronically implanted cannulae and acute infusions allowed animals to act as their own controls and reduced experimental variation caused by intersubject differences. For all infusions the monkey was held gently by a researcher. The cannulae caps and cannula blockers were removed and the site cleaned with 70% ethanol. A sterile injector was inserted into the relevant cannula and saline or drug infused over a period of 2 min. Injectors were left in place for 1 min to allow diffusion of liquid before being removed, clean caps and cannula blockers were applied, and the monkey returned to the homecage for the required pretreatment time. The approach–avoidance sessions were interspersed between baseline reward sessions. Infusions usually occurred twice a week (one saline treatment and one drug treatment) in a randomised order between groups. All drugs were dissolved in advance and frozen as individual aliquots until required. Before use they were thawed and brought up to room temperature. Drug infusions were as follows. 1) Regional inactivation of a brain region via muscimol/baclofen (0.5 μL of 0.1 mM muscimol [Sigma, UK]/1.0 mM baclofen [Sigma, UK]) infusion at a rate of 0.25 μL/min and a pretreatment time of 25 min (“musbac”). 2) Regional overactivation of brain regions by increasing glutamate levels via an infusion of 1 μL of a mixture of the mGlu2/3 receptor antagonist LY341495 (1 ng/μL; Tocris Bioscience, UK), and the GABA_B_ receptor antagonist, CGP52432 (1 ng/μL; Tocris Bioscience, UK), at a rate of 0.5 μL/min, with a 15 min pretreatment time. These receptors both act to limit presynaptic glutamate release, thus their antagonism with this cocktail (“LY/CGP”) acts to increase the amount of glutamate released (Marrocco et al. 2012). 3) Antagonism of pre- and postsynaptic 5-HT1a receptors with WAY100135 (1 μg/μL Tocris, UK; “WAY”) infused at a rate of 0.25 μL/min with a pretreatment time of 25 min. 4) Saline control infusions (see Supplementary Materials and Methods for the order of drug infusions).

### Behavioral and Statistical Analysis

For each daily session, the number of responses made to each side was collected and a bias measure was calculated for each animal. This was the ratio of the number of responses made to the nonpreferred side over the number of responses made to the preferred side (bias = nonpreferred side responses / preferred side responses). A bias measure of 1 indicates animals responded to both sides equally, with increasing bias measures indicating a bias away from the preferred side. As all animals had a slight innate preference for a particular (and thus preferred) side, their normal response bias is <1. As there was individual variation in the overall number of responses that animals made, this bias measure allows the standardization of behavior across animals without the confound of response number variability in the total number of responses. However, as an aggregate function, the bias measure does not allow the interpretation of any bias change observed, that is, a relative shift away from punishment could be indicative of punishment avoidance, an increased preference for the nonpunished/rewarded side, or both. For this reason, we also analyzed the total number of responses made to both the preferred and nonpreferred sides. Additional measures included:

– Latency to respond to the side that was rewarded or punished on the immediately preceding trial (side latencies) and the latency to respond to any side following reward or punishment (general latency).
– Overall numbers of rewards received. However, because a variable VI response schedule was in operation the relationship between numbers of responses and rewards was relatively weak and so a reduction in responding following punishment impacted very little on the total number of rewards obtained (see Supplementary materials and methods). Consequently, the number of responses made on the punished side is a better indicator of sensitivity to punishment than numbers of rewards obtained.

Response data were square-root transformed to avoid violations of the assumptions of ANOVA and analyzed using repeated measures ANOVA in SPSS v22 (IBM, NY, USA) and R (R Core Team, 2016). However, for clarity the data presented in the graphs are not transformed. Factors included condition (2 levels; baseline reward and approach–avoidance), response side (2 levels; preferred and nonpreferred), drug (up to 4 levels including saline, musbac, LY/CGP, and WAY) and area (levels including aHipp, area 32, area 25, and combinations). Because a previous study using this paradigm identified drug effects on both the infused/punished day, and the next day, when only baseline reward was delivered (Clarke et al. 2015), next-day performance was also analyzed. However, all terms involving the factor “day” (infusion or next days) were not significant and the data is therefore not displayed. In addition, covarying the infusion data for the previous days’ performance (day “zero”) did not affect the results (see Supplementary materials and methods).

### Euthanasia and Histology

As described previously (Wallis et al. 2017), all monkeys were sedated with ketamine hydrochloride (Pharmacia and Upjohn, 0.05 mL of a 100 mg/mL solution, i.m.) and humanely euthanased with Euthatal (1 mL of a 200 mg/mL solution, pentobarbital sodium; Merial Animal Health Ltd; i.v.) before being perfused transcardially with 400 mL of 0.1 M PBS, followed by 400 mL of 4% paraformaldehyde fixative over approximately 30 min. The entire brain was then removed and placed in further paraformaldehyde overnight before being transferred to PBS. Prior to sectioning, the brain was placed in 30% sucrose solution for at least 48 h. For verification of cannulae placement, coronal sections (60 μm) of the brain were cut using a freezing microtome, the cell bodies were stained using Cresyl Fast Violet and the sections viewed under a microscope. For each animal, cannula locations were schematized onto drawings of standard marmoset brain coronal sections and composite diagrams were then made to illustrate the extent of overlap between animals.

## Results

All 5 marmosets adopted the optimal strategy to maximize reward delivery by responding to both sides relatively consistently and equally. The total number of responses they made during baseline performance therefore remained stable across the study with an average of 3.21 ± 0.06 responses made per reward (see Supplementary Fig. S1a/b). However, marmosets did also show a slight side bias, tending to respond to one side (preferred side) more than the other (nonpreferred side). The direction of this intrinsic bias varied between monkeys, but also remained relatively stable within individuals across the study (see Supplementary Fig. S1c). Consequently, comparison of the number of responses made on each side (averaged across all brain saline infusions on baseline reward only days) revealed a significant response bias to their preferred side (baseline reward; *t*_4_ = 3.5, *P* = 0.024; Fig. 1*B*). However, this intrinsic bias was eliminated by the introduction of punishment (aversive noise, 0.3 s, 117 dB) on the preferred side as the marmosets no longer biased their responding to that side (after saline treatment; condition_2_ × response side_2_, *F*_1,4_ = 27.477, *P* = 0.006; effect of responses side during Approach–avoidance condition, *t*_4_ = 0.301, *P* = 0.779). This reduction in intrinsic bias as a consequence of punishment on the preferred side was not due to any change in responding on the nonpreferred side, but was due to a trend-level reduction in responding to the preferred side (as shown by analysis of total number of responses to preferred/nonpreferred sides across conditions on the infusion day: nonpreferred, *t*_4_ = −0.409, *P* = 0.703; preferred, *t*_4_ = −2.1, *P* = 0.103). Together these analyses show that the aversive noise induces a purely avoidant response (Fig. 1*B*). This avoidant response did not “carry over” and impact on the next day performance (when punishment was again absent; Fig. 1*B*) as the intrinsic bias returned on the next day. ANOVA revealed that performance on the next day following punishment did not differ from baseline saline conditions on either the infusion day, or the next day (condition_2_ × response side_2_: next day approach–avoidance versus next day baseline, *F*_1,4_ = 1.63, *P* = 0.271; next day approach–avoidance versus infusion day baseline, *F*_1,4_ = 2.58, *P* = 0.18).

**Figure 2.**
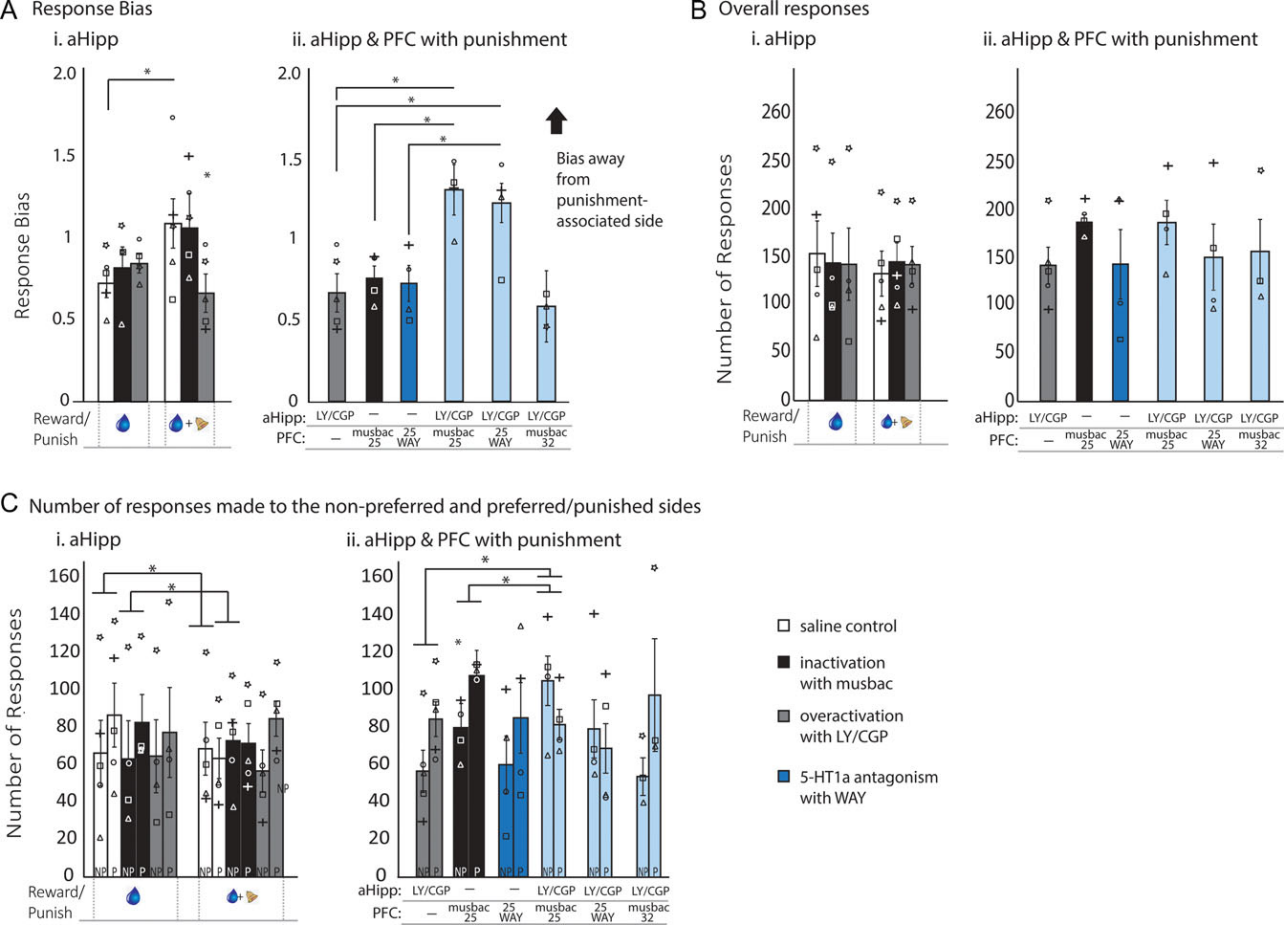
Overactivation of the aHipp abolishes punishment avoidance, but this response is prevented by simultaneous inactivation or 5-HT1a antagonism within area 25. Pharmacological manipulation of the aHipp and combined manipulations of the aHipp and either area 25 or area 32 produced differential effects on responding in the baseline reward (blue drop only) and approach–avoidance (blue drop and bell) conditions. Figures show the drug-induced changes in response bias (the ratio between the number of responses to the monkey’s nonpreferred side and the preferred side), the absolute response number and the relative responding to the preferred (P) and nonpreferred (NP) sides compared with saline controls. Data are represented as mean ± SEM. *P < 0.05. Response bias data is square root transformed for analysis. Inactivation of the aHipp with musbac did not affect responding in either the baseline reward or approach–avoidance condition, but overactivation with LY/CGP decreased punishment avoidance (*A*i). No the alterations were seen in the overall number of responses (*F*s < 1, NS; *n* = 5; *B*i). Analysis of relative responding to the preferred and nonpreferred sides indicated that the alteration in behavior was selectively due to a reduction in responding to the punished side, and not an increase in responding to the nonpreferred side (*C*i). Combining aHipp LY/CGP infusion with simultaneous inactivation of area 25, and to lesser extent antagonism of 5-HT1a receptors within area 25, abolished the reduction in punishment avoidance seen with independent aHipp LY/CGP infusion, area 25 inactivation and area 25 5-HT1a blockade (*n* = 4). The reduction seen after combined aHipp LY/CGP + musbac 25 was due to both a decrease in responding to the preferred/punished side, and an increase in responding to the nonpreferred side (*C*ii). Simultaneous inactivation of area 32 did not alter the effects of aHipp LY/CGP (*n* = 3; *A*ii). No alterations were seen in the overall number of responses (*F* > 1, NS; Bii). Musbac, muscimol/baclofen-mediated inactivation; LY/CGP, LY341495/CGP52432-mediated presynaptic glutamatergic disinhibition; WAY, WAY100135-mediated 5-HT1a antagonism. NP, nonpreferred side; P, preferred side.

### Presynaptic Glutamatergic Disinhibition, but not Inactivation, of the aHipp Reduces Sensitivity to Punishment

Pharmacological inactivation or presynaptic glutamatergic disinhibition of the aHipp had no effect on the monkeys’ preference for their preferred side when responding for baseline reward only. Thus they maintained the same intrinsic response bias regardless of drug treatment. Inactivation of the aHipp with musbac also had no effect on the subsequent punishment bias in the approach–avoidance condition, as they continued to avoid the punished side without increasing their responding to the nonpunished side. In contrast, the infusion of LY/CGP (Fig. 2*A*i) abolished this avoidance response, with the monkeys maintaining their preferential responding to the preferred/punished side as if the punishment was not present (Fig. 2*A*i/*C*i). ANOVA revealed a Condition_2_ × drug_3_ interaction (*F*_2,18.26_ = 4.82, *P* = 0.0208). There was no drug effect in the baseline reward condition (*F*_2,6.5_ = 1.16, *P* = 0.368) but a drug effect in the approach–avoidance condition (*F*_2,8_ = 4.5, *P* = 0.047). There was no effect of inactivation (saline vs. musbac, *t*_4_ = 0.263, *P* = 0.805) but a significant effect of overactivation (saline vs. LY/CGP, *t*_4_ = 5.4, *P* = 0.006) in the approach–avoidance condition. Similarly, ANOVA of the total number of responses to the preferred and nonpreferred sides revealed a condition_2_ × side preference_2_ interaction for saline (*F*_1,4_ = 9.301, *P* = 0.038) and musbac (*F*_1,3_ = 410.7, *P* < 0.001), that was abolished with aHipp LY/CGP (*F*_1,3_ = 1.245, *P* = 0.356), and an overall condition_2_ × drug_3_ × side preference_2_ interaction (*F*_2,18.26_ = 4.82, *P* = 0.0208).

No main effects were seen on the overall number of responses (*F*s < 1, NS; Fig. 2*B*i), the number of rewards obtained (*F*s < 1, NS; see Supplementary), or the latencies to respond after rewarding (largest *F* value = 2.93, all NS) or punishing feedback (largest *F* value = 2.97, all NS; *n* = 5). However, consistent with the lack of punishment avoidance after aHipp LY/CGP, targeted investigation revealed that aHipp LY/CGP treated animals were faster to respond again to the punished side after receiving punishment compared with the saline treated animals (saline vs LY/CGP, *t*_4_ = 4.472, *P* = 0.011). Thus, glutamatergic disinhibition within the primate aHipp abolishes the sensitivity to punishing feedback in approach–avoidance decision making.

### Presynaptic Glutamatergic Disinhibition Within the aHipp Abolishes Sensitivity to Punishing Feedback via 5-HT1a Receptors in Area 25, but not Area 32

Correlative human imaging studies and rodent electrophysiological studies have both highlighted the importance of communication between the aHipp and the mPFC in the regulation of negative emotion (Adhikari et al. 2010; Hamilton et al. 2011; Padilla-Coreano et al. 2016). Therefore we determined whether the abolition of punishment–avoidance by aHipp LY/CGP was dependent upon interactions with the mPFC. The hippocampus is known to modulate activity in both areas 25 and 32 via direct aHipp–mPFC projections, so we prevented any aHipp-mediated effects in these regions by combining aHipp LY/CGP with inactivation of either area 25 or 32. The reduction in punishment avoidance seen with aHipp LY/CGP treatment alone was abolished with simultaneous inactivation of area 25. Animals avoided the punished side more than in saline control conditions and responded more to the nonpreferred side. Simultaneous aHipp LY/CGP infusion and area 32 inactivation did not have this effect and animals continued to respond to the punished side as if with aHipp LY/CGP alone. This suggests that an aHipp–area 25 circuit acts to regulate approach–avoidance performance while an aHipp–area 32 circuit does not (main effect of area 25 manipulations on bias: *F*_3,9_ = 13.03, *P* = 0.001; aHipp LY/CGP + musbac 25 vs. aHipp LY/CGP; *t*_3_ = 4.6, *P* = 0.019; aHipp LY/CGP + musbac 25 vs. musbac 25; *t*_3_ = 9.46, *P* = 0.003; aHipp LY/CGP + musbac 25 vs. 25 saline; *t*_3_ = 10.3, *P* = 0.002). There was also a main effect of area 25 manipulations on total responses on the preferred and nonpreferred sides: drug_3_ × side preference_2_, *F*_2,6_ = 16.343, *P* = 0.004; aHipp LY/CGP + musbac 25 vs aHipp LY/CGP; drug_2_ × side preference_2_, *F*_1,11.9_ = 9.5, *P* = 0.009, preferred side, *t*_4_ = 3.42, *P* = 0.749, nonpreferred side, *t*_4_ = 3.5, *P* = 0.025; aHipp LY/CGP + musbac 25 vs. musbac 25, drug_2_ × side preference_2_, *F*_1,9.76_ = 19.9, *P* = 0.0013, preferred side, *t*_3_ = 3.409, *P* = 0.042, nonpreferred side, *t*_3_ = 2.89, *P* = 0.063; aHipp LY/CGP + musbac 25 vs. 25 saline, drug_2_ × side preference_2_, *F*_1,3_ = 61.8, *P* = 0.004, preferred side, *t*_3_ = 2.1, *P* = 0.126, nonpreferred side, *t*_3_ = 4.3, *P* = 0.022. There was no main effect of area 32 manipulations on bias: *F*_3,6_ = 2.1, *P* = 0.2: aHipp LY/CGP + musbac 32 vs. aHipp LY/CGP; *t*_2_ = 0.452, *P* = 0.696; aHipp LY/CGP + musbac 32 vs. musbac 32; *t*_2_ = 2.1, *P* = 0.182; or total responses on the preferred and nonpreferred sides, *F* < 1, NS (see Fig. 2*A*ii/*C*ii).

Given the high concentration of 5-HT1a receptors in area 25, we also investigated the contribution of 5-HT1a receptors to the aHipp–area 25 circuit using WAY in area 25 instead of musbac, and replicated the return of the punishment avoidance that was seen with aHipp LY/CGP + 25 musbac. (Main effect of area, *F*_1,3.3_ = 19.7, *P* = 0.016; aHipp LY/CGP vs aHipp LY/CGP + area 25 WAY, *t*_3_ = 4.5, *P* = 0.020.) This suggests that an aHipp–area 25 circuit (involving area 25 5-HT1a receptors) acts to regulate approach–avoidance performance while an aHipp–area 32 circuit does not (Fig. 2*A*ii).

No alterations were seen in the overall number of responses (*F* > 1, NS; Fig. 2*B*ii), or in the latencies to respond after punishment (largest *F* value = 4.02, all NS) or reward (*F*s < 1, NS). However, targeted investigation revealed that the combined aHipp LY/CGP + area 25 WAY abolished the faster trial latencies seen after punishment with aHipp LY/CGP alone (*t*_3_ = 3.392, *P* = 0.046). Although aHipp LY/CGP + area 25 musbac increased responding compared with aHipp LY/CGP alone, this was not significant (number of responses, *t*_3_ = 2.641, *P* = 0.07), but was nevertheless sufficient to cause a corresponding increase in the number of rewards obtained (*t*_3_ = 3.332, *P* = 0.045, see Supplementary).

### Inactivation and 5-HT1a Antagonism of Area 25 Produced Opposing Effects to Area 25 Overactivation on Responsivity to Punishment

Whilst the findings so far suggest that the reduction in punishment sensitivity following glutamatergic disinhibition within the aHipp is dependent upon interactions between the aHipp and area 25, the effects could have been due to the summation of independent effects of aHipp and area 25 manipulations. Therefore, we also selectively inactivated area 25 and 32 without aHipp activation on the baseline reward and approach–avoidance conditions. We also blocked 5-HT1a receptors in area 25 with WAY.

**Figure 3.**
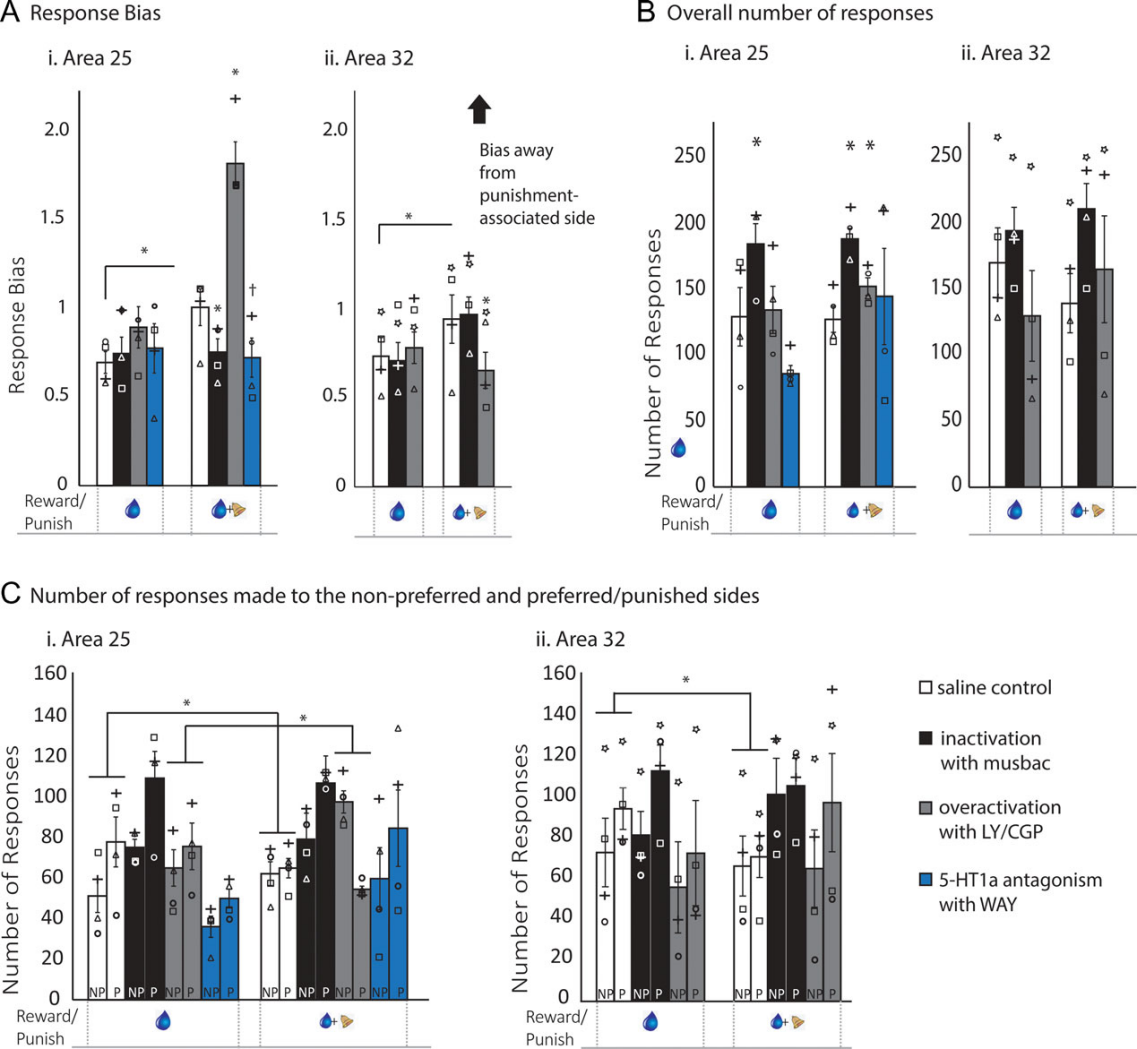
Activity within area 25 and 32 has opposing roles in the regulation of punishment avoidance. Independent pharmacological manipulation of areas 25 and 32 produced differential effects on responding in the baseline reward (blue drop only) and approach–avoidance (blue drop and bell) conditions. Figures show the drug-induced changes in response bias, the absolute response number and the relative responding to the preferred and nonpreferred sides compared with saline controls. Data are represented as mean ± SEM. ^†^Trend effect of manipulation. *P < 0.05. Response bias data is square root transformed for analysis. Inactivation or 5-HT1a antagonism within area 25 prevented the avoidance of punishment seen after saline infusion, while overactivation enhanced the avoidance of punishment (*n* = 4). Drug infusions did not affect performance in the baseline reward condition but did affect the approach–avoidance condition (*A*i). Within the approach–avoidance condition, punishment avoidance was enhanced with LY/CGP and abolished with musbac. While LY/CGP caused this by both reducing responding to the preferred/punished side and increasing responding on the nonpreferred side, musbac selectively increased responding to the preferred side only (*C*i). A similar trend towards abolition was seen with 5-HT1a antagonism. Manipulation of area 25 also altered the overall number of responses made (*B*i). Inactivation of area 25 increased the number of responses in both conditions, while 5-HT1a antagonism had no effect. Area 25 LY/CGP increased the number of responses in the approach–avoidance condition only. Overactivation and inactivation of area 32 also had different effects on response bias in the 2 feedback conditions (*n* = 4; *A*ii). Infusions of musbac or LY/CGP did not affect performance in the baseline reward condition, and inactivation was without effect in the approach–avoidance condition. However, area 32 overactivation reduced the response bias to punishing feedback. No alterations were seen in the overall number of responses (*F*s < 1, NS; *B*ii), and the response bias alteration was caused by a selective increase in responding to the preferred/punished side (*C*ii). Musbac, muscimol/baclofen-mediated inactivation; LY/CGP, LY341495/CGP52432-mediated presynaptic glutamatergic disinhibition; WAY, WAY100135-mediated 5-HT1a antagonism. NP, nonpreferred side; P, preferred side.

As with the aHipp, pharmacological manipulation of area 25 with musbac, LY/CGP or WAY had no effect on the monkeys’ intrinsic side bias during baseline reward sessions, as they maintained the same response bias after all drug treatments (Fig. 3*A*i/*C*i, left). However, these manipulations had different effects on approach–avoidance bias performance in the presence of punishment. Thus, repeated measures ANOVA revealed an interaction effect of drug_4_ × condition_2_ (*F*_3,9_ = 16.062, *P* = 0.001), with effects of drug in the approach–avoidance condition (*F*_3,9_ = 47.7 *P* < 0.0001) but not the baseline reward condition (*F*_3,9_ = 0.446 *P* = 0.726). Inactivation of area 25 with muscimol/baclofen (musbac) eliminated the monkeys’ response bias away from punishment, and they continued to respond to the punished side as if no punishment was present (saline vs. musbac, *t*_3_ = 3.631, *P* = 0.036, Fig. 3*A*i, right), comparable to that seen following LY/CGP into the aHipp. Similarly, 5-HT1a antagonism within area 25 with WAY also tended to abolish punishment avoidance although this did not reach significance (saline vs. WAY, *t*_3_ = 2.274, *P* = 0.107). Conversely, overactivation of area 25 with LY/CGP caused an increase in punishment avoidance (saline vs. LY/CGP, *t*_3_ = 5.847, *P* = 0.01, Fig. 3*A*i, right).

Analysis of total responding to the preferred and nonpreferred sides revealed that the lack of punishment avoidance shown by both musbac- and WAY- treated animals was due to a failure to show the reduced responding to the preferred/punished side seen after saline treatment. In contrast, the enhanced punishment avoidance seen in the LY/CGP animals was due to them both reducing their responding on the preferred/punished side and increasing their responding to the nonpreferred side. Repeated measures ANOVA revealed an interaction effect of drug_4_ × condition_2_ × side preference_2_ (*F*_3,33_ = 2.7, *P* = 0.035), with condition_2_ × side preference_2_ interactions for both saline and LY/CGP, but not musbac or WAY (saline, *F*_1,3_ = 11.48, *P* = 0.043; LY/CGP, *F*_1,3_ = 74.5, *P* < 0.003; musbac, *F* < 1, NS; WAY, *F*_1,3_ = 2.297, *P* = 0.227). Specifically, the tendency to bias responding to the preferred side in the baseline reward condition was abolished by the introduction of punishment after saline treatment, enhanced or unchanged by musbac and WAY treatment, and reversed by LY/CGP treatment (saline: baseline reward, *t*_3_ = 3.7, *P* = 0.034, approach–avoidance, *t*_3_ = 0.409, *P* = 0.71; musbac: baseline reward, *t*_3_ = 2.8, *P* = 0.066, approach–avoidance, *t*_3_ = 3.66, *P* = 0.035; WAY: baseline reward, *t*_3_ = 1.8, *P* = 0.165, approach–avoidance, *t*_3_ = 2.05, *P* = 0.133; LY/CGP: baseline reward, *t*_3_ = 3.04, *P* = 0.056, approach–avoidance, *t*_3_ = 7.1, *P* = 0.006; Fig. 3*C*i).

Thus, while normal activity and 5-HT1a function within area 25 are required to mediate the avoidance of punishment in approach–avoidance decision making, overactivation increases responsivity to punishment, indicating that area 25 activity has bidirectional effects on punishment responsivity. Area 25 activity, and 5-HT1a function in this area are therefore required to mediate the avoidance of punishment in approach–avoidance decision making, and have similar effects to aHipp LY/CGP.

Besides influencing the punishment bias, pharmacological manipulation of area 25 also had effects on the total numbers of responses (main effect of drug_3_, *F*_3,9_ = 11.171, *P* = 0.002). Inactivation of area 25 increased the total responses made across both baseline reward (*F*_3,9_ = 13.436, *P* = 0.001) and approach–avoidance conditions (*F*_3,9_ = 13.436, *P* = 0.001; Fig. 3*B*i). In contrast, 5-HT1a antagonism did not alter the number of responses made in either the baseline reward (*t*_3_ = 2.289, *P* = 0.106) or the approach–avoidance conditions (*t*_3_ = 0.292, *P* = 0.789), whilst area 25 overactivation increased the total number of responses made in the approach–avoidance condition only (baseline reward condition, *t* < 1, NS; approach–avoidance, *t*_3_ = 6.108, *P* = 0.009; Fig. 3*B*i). Please see the Supplementary materials and methods for details of changes in rewards received.

Trial and general latencies to respond after rewarding feedback were unaltered (all area × drug interactions NS, largest *F* value = 1.23). General latencies to respond after punishment were unaffected by drug treatment (for all terms involving drug, *F*s < 1, NS), while a marginal effect of drug on trial latencies after punishment did not reach significance (*F*_2,9_ = 3.419, *P* = 0.078). A focused analysis of the trial latencies of those manipulations that did show an alteration in bias revealed that while area 25 musbac and area 25 WAY both accelerated responding, and area 25 LY/CGP slowed responding, these effects were not significant (all NS, largest *t* = 1.03).

### Overactivation of Area 32 Altered Approach–Avoidance Performance

Manipulation of area 32 revealed effects that differed from the manipulation of area 25 (area_2_ × drug_3_ × condition_2_, *F*_2,32.15_ = 15.537, *P* = 1.9 × 10^e-5^; Fig. 3*A*ii). In area 32, neither inactivation nor overactivation affected performance in the baseline reward condition with animals continuing to bias their responding towards their preferred/punished side. Only overactivation impacted performance in the approach–avoidance condition, acting to reduce punishment avoidance. ANOVA revealed a main effect of drug_3_ × condition_2_ for area 32 (*F*_2,15_ = 5.736, *P* = 0.0141), with no main effect of the baseline reward condition (*F*_2,6_ = 0.441, *P* = 0.663; Fig. 3*A*ii). Inactivation had no effect on the monkeys’ response bias in the approach–avoidance condition where they continued to avoid the punished side (main effect of approach–avoidance condition; *F*_2,6_ = 6.7, *P* = 0.03, but no effect of saline vs. musbac, *t*_3_ = 0.336, *P* = 0.759). However, area 32 overactivation reduced the bias away from punishing feedback (saline vs. LY/CGP: approach–avoidance, *t*_3_ = 5.485, *P* = 0.012).

No alterations were seen in the overall number of responses (*F*s < 1, NS; Fig. 3*B*ii), and while the total responding on the preferred and nonpreferred sides differed from area 25 (area_2_ × drug_4_ × condition_2_ × side preference_2_, *F*_2,67.85_ = 12.9, *P* = 0.025), there was no drug_4_ × condition_2_ × side preference_2_ interaction within area 32 (*F*_2,33_ = 1.21, *P* = 0.31; although targeted investigation revealed a condition_2_ × side preference_2_ interactions for saline only, *F*_1,3_ = 12.9, *P* = 0.037). Like area 25, manipulations of area 32 did not alter the trial latencies to respond after reward, or the general latencies to respond after either rewarding or punishing feedback (all terms involving area NS, largest *F* value = 1.2). Within area 32, the effects of drug treatment on the trial latencies after punishment differed from those seen in area 25 (*F*_2,14.6_ = 4.46, *P* = 0.031). However, there was no main effect of drug within area 32 (*F*_2,6_ = 4.73, *P* = 0.059), and targeted investigations of those manipulations that caused alterations in bias also revealed no alterations in latency (saline vs LY/CGP, *t*_3_ = 2.267, *P* = 0.11). See the Supplementary for details of changes in rewards received.

In summary, activity within area 32 appears to act in opposition to area 25.

### The aHipp Shows Interaction With Area 25, but not Area 32, During the Regulation of Approach–Avoidance Performance

Given that area 25 inactivation and area 25 5-HT1a antagonism caused similar insensitivities to punishment as aHipp LY/CGP, we were interested in whether there was evidence for an interaction between the aHipp and areas 25 and 32. For each drug treatment, we therefore classified all three brain regions as being at their baseline level of activity (no infusion or saline vehicle infusion), activated (LY/CGP), or inactivated (musbac). For example, after aHipp LY/CGP treatment, aHipp is “activated”, area 25 is at “baseline” and area 32 is at “baseline.” This allowed us to examine whether the effects associated with alterations in activity within one area, were dependent upon the level of activity in another area.

To investigate aHipp–area 25 interactions we performed a two-way ANOVA on the response bias data with factors of “aHipp activity state” (activation or baseline), and “area 25 activity state” (inactivation or baseline), on sessions for which area 32 activity was at baseline. This revealed a significant interaction between the activity states of the aHipp and area 25 (*F*_1,17.9_ = 42.6, *P* = 3.8 × 10^−6^). Subsequent post hoc ANOVAs sought to compare the directionality of this interaction, and revealed that the combined aHipp activation + area 25 inactivation treatment abolished the reductions in punishment avoidance seen after either aHipp activation or area 25 inactivation alone (main effect of treatment, *F*_2, 6.34_ = 15.3, *P* = 0.003; where aHipp LY/CGP + musbac 25 vs. aHipp LY/CGP, *t*_3_ = 4.6, *P* = 0.019; aHipp LY/CGP + musbac 25 vs. musbac 25; *t*_3_ = 9.46, *P* = 0.003). This confirms that the activity in each structure depends upon the activity in the other and reveals the importance of interaction between these structures for the regulation of punishment sensitivity. The same interaction effect between activity states within aHipp and area 25 was also seen when substituting “area 25 5-HT 1a activity state” for “area 25 activity state” (*F*_1, 18.1_ = 32.047, *P* = 2.2 × 10^−5^). Post hoc ANOVA confirmed that combined aHipp activation + area 25 5-HT 1a inactivation replicated the return of the punishment avoidance that was seen with combined aHipp activation + area 25 inactivation. (ANOVA revealed a main effect of aHipp LY/CGP + 25 WAY vs. aHipp LY/CGP, *F*_1, 3.3_ = 19.7, *P* = 0.016; *n* = 4.) However, no such relationship was seen between the aHipp and area 32 (*F*_1, 16.8_ = 0.0001, *P* = 0.99).

This suggests that activity within an aHipp–area 25 circuit (involving area 25 5-HT1a receptors) acts to regulate approach–avoidance performance while an aHipp–area 32 circuit does not (Fig. 2*A*ii).

## Discussion

These results reveal the crucial, dissociable roles played by the aHipp–mPFC circuitry, and its individual components, in modulating instrumental approach–avoidance decisions. Animals normally avoided punishment, but enhancement of presynaptic glutamate release in the aHipp decreased punishment avoidance, while inactivation had no effect. This reduction in punishment avoidance was abolished by simultaneous inactivation (induced by GABA_A&B_ receptor activation) of area 25, but not area 32, revealing the differential interaction of aHipp with area 25 (but not area 32) in the regulation of approach–avoidance decision making by punishing feedback. Of note, area 25 inactivation alone decreased punishment avoidance, the opposite effect to that seen when the same manipulation was combined with aHipp overactivation while overactivation of this same area increased punishment avoidance. Thus, although aHipp overactivation and area 25 inactivation both independently decrease punishment avoidance, when occurring simultaneously their independent effects are abolished and behavior normalized, indicating the importance of communication between the two areas for their regulation of avoidance. In contrast, area 32 inactivation alone had no impact on approach–avoidance suggesting that under normal task conditions, this region is not recruited. However, the finding however, that its overactivation potentiated punishment avoidance, indicates that it can regulate performance when required.

### Consideration of Tasks Used to Study Approach–Avoidance Behavior

The majority of rodent approach–avoidance paradigms use unlearned, innate cues to measure unconditioned approach–avoidance behaviors in ethological situations (such as the elevated plus maze and open field) where in most cases there is no explicit reward or punishment. In contrast, the present paradigm measures instrumental approach–avoidance decision making in which animals learn that a particular rewarded action has become associated with unpredictable punishment. The latter is highly relevant to many of the cost-benefit decisions that are experienced in the everyday life of humans, and importantly, are impaired in affective disorders (Dickson and MacLeod 2004; Dickson 2006). Of particular relevance, a similar learned approach–avoidance paradigm has also been developed for rodents to capture such decision making (Schumacher et al. 2016). However, the use of Pavlovian cues to signal the presence of reward or punishment in that rodent version of the task adds an extra layer of control that is not present in the current paradigm. Such differences need to be taken into account when comparing the present findings to the existing literature.

### Hippocampal Contributions to Approach–Avoidance Behavior and Interaction With Area 25

Inactivation of aHipp had no effect in the current approach–avoidance paradigm, despite the well-documented effects of lesions or sclerosis of the aHipp in reducing avoidance and increasing approach in a variety of threatening situations in rodents, primates and humans (Chudasama et al. 2008, 2009; Bach et al. 2014; O’Neil et al. 2015; Schumacher et al. 2016; Korn et al. 2017). It is unlikely that the task did not induce enough anxiety, and therefore decision complexityu, to recruit the aHipp (Aupperle and Paulus 2010; Aupperle et al. 2015) as we have previously demonstrated that punishment avoidance in this paradigm is ameliorated by anxiolytic treatment with benzodiazepines (Clarke et al. 2015). Thus like human, rodent, and macaque approach–avoidance paradigms (Amemori and Graybiel 2012; Calhoon and Tye 2015), anxiety is contributing to the approach–avoidance decision. An alternative explanation may lie in the marked differences in the tasks used to study approach–avoidance behavior, as described above. For example, the anxiolytic effect of vHipp lesions in the rodent approach–avoidance task with pavlovian cues may be a consequence of the Pavlovian component of the task, rather than the approach–avoidance component (O’Neil et al. 2015; Schumacher et al. 2016). No such Pavlovian component was present in the current task. Indeed, direct comparison of select vHipp manipulations on decision making has revealed contradictory effects between Pavlovian cued approach–avoidance paradigms versus anxious responding in a light/dark box (Schumacher et al. 2018). Thus, until there is a better understanding of the precise contribution of the aHipp to such tasks, it is difficult to explain the lack of aHipp inactivation effects in the current study.

Further insight into the role of the aHipp also needs to take into account potentially distinct contributions of the aHipp subregions. In the majority of rodent vHipp studies discussed so far (reviewed by Barkus et al. 2010; Ito and Lee 2016), manipulations have affected all the main anatomical subregions (dentate gyrus and CA cell fields). Similarly, the large hippocampus-encompassing voxel used in imaging studies to implicate the aHipp in approach–avoidance behavior precludes the identification of individual subregions (Bach et al. 2014). These subregions are functionally heterogeneous, however, and although a few studies have investigated their individual contributions to approach–avoidance and anxious behavior, the findings are not consistent (Kheirbek et al. 2013; Weeden et al. 2015; Jimenez et al. 2018). Of particular relevance to the current findings, lesions of the ventral CA1 and CA3 produce opposing effects in the Pavlovian cued rodent approach–avoidance paradigm, with CA1 inactivation enhancing avoidance of a “conflict” cue and CA3 inactivation reducing it (Schumacher et al. 2018). This indicates that manipulating distinct regions of the vHipp can have effects that differ markedly from the compound effect of targeting the whole vHipp. Histological assessment of the location of the current aHipp cannulae indicates that they primarily targeted the CA3 and CA2 cell fields, rather than the CA1 or dentate gyrus, which may also explain why the inactivation effects in the present study differed from those targeting the whole aHipp. Further studies are therefore required to elucidate more precisely the differential involvement of hippocampal subregions in learned non-cued approach–avoidance behavior.

Nevertheless, the novel finding that overactivation of the primate aHipp interacts with area 25 (but not area 32) to modulate punishment sensitivity during approach–avoidance decision making is consistent with the connectivity patterns of the aHipp, which projects more densely to area 25 than area 32 (Barbas and Blatt 1995; Aggleton et al. 2015). It is also consistent with recent evidence for aHipp–25 and not aHipp–32 interactions in high trait anxious marmosets (Zeredo et al. 2019) Importantly, it provides causal evidence for the proposed links between the aHipp and area 25 in the etiology and amelioration of depressive and anxious states from correlative imaging studies (Dickie et al. 2011; Hamilton et al. 2011; Treadway et al. 2015). It also supports network models of depression that highlight the importance of area 25 in mediating the interaction between higher cognitive areas, subcortical structures and the hippocampus (Seminowicz et al. 2004). Although the primate neurophysiology of the aHipp–mPFC circuitry is unknown, rodent hippocampal afferents synapse on both pyramidal cells and inhibitory interneurons within a large multiregion zone encompassing the mPFC, and their stimulation results in an initial pyramidal activation followed by a long-lasting inhibition (Adhikari et al. 2010, 2011; Carreno et al. 2016). Thus, the aHipp may normally inhibit area 25. Moreover, the findings in the present study show that one such facilitator of this hippocampal communication within area 25 is the inhibitory 5-HT1a receptor. Certainly there is evidence that within the hippocampus, 5-HT1a and GABA_B_ receptors share the same inhibitory potassium channels (Andrade et al. 1986), and 5-HT1a strongly regulates negative emotional responding (Tsetsenis et al. 2007). Thus the aHipp may modulate 5-HT1a function within area 25, and serotonergic signaling via 5-HT1a receptors is a strong candidate for synchronisation within an mPFC–hippocampal circuit (Puig and Gener 2015).

### Independent Contributions of Subgenual and Perigenual Anterior Cingulate Cortex to Approach–Avoidance

Like aHipp overactivation, area 25 inactivation reduced sensitivity to punishment in a manner consistent with previous findings in marmosets in which area 25 inactivation reduced the behavioral and cardiovascular correlates of conditioned fear (Wallis et al. 2017). Furthermore, the increased sensitivity to punishment seen after increased activity within area 25 may provide a causal link between the increased sensitivity to negative feedback seen in negative (depressed) mood states and the association of such states with excessive activity in a subgenual region of the anterior cingulate that includes area 25 (Aupperle and Paulus 2010; Hamani et al. 2011; Trew 2011; Roiser and Sahakian 2013). It is also consistent with the heightened reactivity to uncertain threat in the form of an unknown human followng area 25 overactivation in marmosets (Alexander et al. 2019). As area 25 contains predominantly “negative-encoding” neurons that are activated more by punishment than reward (Monosov and Hikosaka 2012), one explanation for these convergent findings is that overactivity of area 25 enhances the impact of punishment by promoting a negative interpretation of feedback, while inactivation inhibits these neurons to reduce both the corresponding “negative” signal, and the impact of punishment. The inhibition of a negative signal may also have contributed to the overall increase in total responding in both baseline reward and approach–avoidance punishment sessions after area 25 inactivation. As sessions with punishment w randomly interleaved between sessions of baseline reward, the ever-present potential for punishment may normally act to suppress responding, even on baseline reward sessions. By inhibiting a negative “punishment”-related signal, inactivating area 25 may therefore disinhibit responding, as was observed.

However, the finding that both area 25 inactivation and aHipp overactivation individually reduce punishment avoidance, but their combination abolishes this reduction, not only indicates that mPFC–Hipp communication is important for negative emotion regulation (Adhikari et al. 2010; Dickie et al. 2011; Padilla-Coreano et al. 2016), but also suggests that disruption of this communication may recruit other areas that can modulate punishment sensitivity, and whose action was previously masked by activity within area 25. Indeed, an unmasked role for a different aHipp projection area may explain why the combination of aHipp LY/CGP + area 25 inactivation not only abolished the reduction in punishment avoidance seen with each manipulation independently, but actually appeared to enhance avoidance. Other projection structures previously implicated in approach–avoidance decision making include the amygdala, the striatum, the OFC, and the vlPFC (Gourley et al. 2013; Clarke et al. 2015; Winstanley and Floresco 2016; Piantadosi et al. 2017). However, whereas an OFC–amygdala circuit was shown to modulate long-lasting mnemonic effects of punishment avoidance, vlPFC inactivation immediately enhanced punishment avoidance. Given that the aHipp also projects to vlPFC, this alternative projection may also be implicated, although this remains to be investigated. Consequently, care may need to be taken when therapeutically reducing area 25 activity (Mayberg et al. 2005) to ensure that activity is normalized rather than abolished, as area 25 activity is clearly an important effector of adaptive communication from the hippocampus.

In contrast to the consistent anxiolytic and anxiogenic effects of area 25 inactivation and overactivation respectively in the Pavlovian conditioned fear and approach–avoidance decision-making tasks, the effects of manipulations of area 32 were not consistent across these paradigms. While area 32 did regulate the behavioral and cardiovascular correlates of conditioned fear, as shown by pronounced increases following inactivation (Wallis et al. 2017), it was not recruited by the current approach–avoidance task, as shown by the lack of an inactivation effect. Nevertheless, the decreased avoidance of punishment seen after area 32 overactivation indicates that area 32 does play a regulatory role under certain conditions. It is unlikely that the differences in the contribution of area 32 inactivation to these two paradigms is due to different levels of fear, as both paradigms used the same volume and duration of aversive noise. It is also unlikely that the effects of area 32 manipulation depend on the presence of Pavlovian cues signaling the arrival of punishment such as in the conditioned fear paradigm, since overactivation of area 32 still reduced punishment avoidance in the approach–avoidance paradigm despite the lack of Pavlovian cues. Although area 32 has previously been investigated in primates, this was in a study that stimulated a subzone of area 32 cells that have previously been shown to encode “negative” subjective motivational value. As might be predicted, such stimulation induced punishment avoidance in an approach–avoidance paradigm (Amemori and Graybiel 2012), the opposite effect to that seen here. However, this finding is hard to compare to our pharmacological inactivation of the whole area 32, which contains a mixture of positive and negative encoding cells (Amemori and Graybiel 2012).

Other key differences between the approach–avoidance and conditioned fear tasks may provide alternative explanations for their differential sensitivity to area 32 inactivation. The first is controllability. Whereas the approach–avoidance paradigm allows animals to bias their responding to avoid the punishment, the conditioning paradigm does not permit such control. The controllability of stressors can be a key determinant of their behavioral sequelae, with the presence of control preventing many of the outcomes that occur when the stressor is uncontrollable – for example, exaggerated fear. In rodents, detection of such control depends upon activity in the mPFC (Amat et al. 2005), but these studies have not fully differentiated between the IL and PL mPFC subregions, and the contribution of their putative primate homologs (areas 25 and 32), to controllability are unknown. The second is the role of goal directed and/or stimulus–response behaviors. The IL and PL also contribute to the balance between goal-directed actions and stimulus–response habits, which are important for approach–avoidance performance, but not fear conditioning. Certainly lesions of the putative homolog of area 25, namely IL in rodents, reduce habitual responding, leaving responses primarily goal directed, and lesions of the putative homolog of area 32, the rodent PL, disrupt goal-directed responding to leave behavior under habitual control (Coutureau and Killcross 2003; Haddon and Killcross 2011). In this case, however, inactivation of area 25 would be expected to increase sensitivity of responding to changes in the goal, rather than reduce it as seen here, and inactivation of area 32 would be expected to decrease sensitivity of responding to changes in goal, rather than have no effect. Furthermore, while hippocampal lesions have been associated with an increase in habitual behavior (Vilà-Balló et al. 2017), the present aHipp inactivations had no effect. Nevertheless, future studies should explicitly address the contributions of controllability and instrumental learning to performance of these tasks.

To conclude, we have shown that dysregulation of aHipp–area 25 circuitry, but not aHipp–area 32 circuitry, reduces marmosets’ tendency to avoid punishment in an approach–avoidance task. We have also differentiated the selective contribution of areas 25, 32, and aHipp. These results provide insight into how dysfunction within these particular brain regions can contribute to distinct components of the altered decision making seen in affective disorders. By demonstrating the importance of the aHipp–area 25 circuit in particular, they also highlight the importance of investigating the role of circuits rather than individual structures in the control of behavior and emotion, as circuit-level modulation may not reflect the cumulative effects of targeting individual structures.

## Supplementary Material

bhz015_Clarke_Supplementary
