# Supplementary material for: Hippocampal Interaction With Area 25, but not Area 32, Regulates Marmoset Approach–Avoidance Behavior
Source: Cereb Cortex. 2019 Feb 23;29(11):4818–30. doi: 10.1093/cercor/bhz015 (PMC6917514; doi:10.1093/cercor/bhz015)

**Supplementary Materials and Methods**

**Small variations in baseline performance do not impact upon the drug effects**

The animals’ baseline performance was relatively stable in terms of both response number and spatial bias (see Figure S1). However to exclude the possibility that minor variations in baseline performance were influencing how the drug infusions altered behaviour we also re-analysed our data to include the previous days performance as a covariate. However repeated measures ANOVA covarying the infusion day with the previous days bias (calculated in the same way as the infusion day regardless of the preferred or non-preferred sides) did not alter the statistical significance of our findings.

Thus covariate analysis of area 25 and 32 data incorporating factors of condition (reward only or approach/avoidance), area (25 or 32) and drug (saline, musbac or LY/CGP) with the pre-infusion days data revealed main effects (condition_2_ x drug_3_ x area_2_, F_2,31.08_ = 16.78, P = 1.14^e-5^; condition_2_, F_1,31.2_ = 16.31, P = 0.00032; area_2_, F_1,34.7_ = 4.72, P = 0.037; Drug_3_, F_2,31.05_ = 20.572, P = 2.044^e-6^) that did not statistically differ from the non-covaried effects (condition_2_ x drug_3_ x area_2_, F_2,32.15_ = 15.57, P = 1.9^e-5^; condition_2_, F_1,32.15_ = 14.06, P = 0.00069; area_2_, F_1,34.8_ = 8.2, P = 0.0069; Drug_3_, F_2,32.15_ = 20.278, P = 2.011^e-6^). Similarly, analysis of the hippocampal manipulation effects revealed no difference with or without covariation when looking at either the hippocampus alone (main effect of condition_2_ x drug_3_: with covariation, F_2,21_ = 4.31, P = 0.027; without covariation, F_2,18.263_ = 4.92, P = 0.02), the hippocampal-mPFC interactions (main effects of area_3:_ with covariation, F_8,10.88_ = 8.18, P = 0.005; without covariation, F_4,19.9_ = 8.18, P = 0.00045), or the hippocampal-area 25 5-HT1a interactions (main effects of area. without covariation, F_1,3.35_ = 19.73, P = 0.017; with covariation, F_1,2.4_ = 24.65, P = 0.026).

**Table 1. Infusion order**

The order of infusions in each monkey was counterbalanced according to area, condition and drug treatment. This study also included an approach/approach condition, but these infusions/data are not shown there.

**Table 1**

|  | Monkey | | | | |
| --- | --- | --- | --- | --- | --- |
| Infusion number | 1 | 2 (32 only) | 3 | 4 | 5 (25 only) |
| 1 | Saline, BL reward, Area 25 | Saline, BL reward, Area 32 | Musbac, BL reward, Area 25 | Saline, BL reward, Area 32 | Saline, BL reward, Area 25 |
| 2 | Saline, App/App, Area 25 | Musbac, App/Av, Area 32 | Musbac, App/Av, Area 25 | Saline, App/Av, Area 25 | Saline, BL reward, aHipp |
| 3 | Musbac, BL reward, Area 25 | Saline, App/Av, Area 32 | Saline, App/Av, Area 25 | Saline, BL reward, Area 32 | LY/CGP, BL reward, aHipp |
| 4 | Saline, App/Av, Area 25 | LY/CGP, App/Av, Area 32 | LY/CGP, App/Av, Area 25 | Musbac, App/Av, Area 25 | Saline, App/Av, aHipp |
| 5 | LY/CGP, App/Av, Area 25 | Musbac, BL reward, Area 32 | Saline, BL reward, Area 25 | Musbac, App/Av, Area 32 | Musbac, BL reward, Area 25 |
| 6 | LY/CGP, BL reward, Area 25 | Musbac, BL reward, aHipp | LY/CGP, BL reward, Area 25 | Saline, BL reward, Area 25 | LY/CGP, App/Av, aHipp |
| 7 | Musbac, App/Av, Area 25 | Saline, App/Av, aHipp | LY/CGP, App/Av, aHipp | Musbac, BL reward, Area 25 | Musbac, BL reward, aHipp |
| 8 | Saline, App/Av, Area 32 | Musbac, App/Av, aHipp | Saline, App/Av, aHipp | LY/CGP, BL reward, Area 25 | 5HT1a area 25, LY/CGP aHipp, App/Av, |
| 9 | \| Musbac, App/Av, Area 32 \| \| --- \| | LY/CGP, App/Av, aHipp | Musbac, App/Av, aHipp | Musbac, App/Av aHipp | Musbac, App/Av aHipp |
| 10 | Musbac, App/Av, aHipp | LY/CGP, BL reward, Area 32 | Musbac Area 25, LY/CGP aHipp, App/Av, | Saline, App/Av aHipp | Musbac, App/Av, Area 25 |
| 11 | Saline, BL reward, Area 32 | LY/CGP BL reward, aHipp | 5HT1a area 25, LY/CGP AHipp, App/Av, | LY/CGP, App/Av, aHipp | Musbac area 25, LY/CGP aHipp, App/Av |
| 12 | Saline, App/Av, aHipp | Saline, BL reward, aHipp | Saline, BL reward, Area 32 | Musbac Area 25, LY/CGP aHipp, App/Av | \| LY/CGP, App/Av, Area 25 \| \| --- \| |
| 13 | LY/CGP, App/Av, aHipp | Musbac Area 32 & LY/CGP aHipp, App/Av | LY/CGP, BL reward, Area 32 | 5HT1a area 25, LY/CGP aHipp, App/Av | Musbac, App/Av, Area 25 |
| 14 | Musbac Area 25, LY/CGP aHipp, App/Av, |  | LY/CGP, App/Av, Area 32 | Musbac, BL reward, Area 32 | LY/CGP, BL reward, Area 25 |
| 15 | 5HT1a area 25, LY/CGP aHipp, App/Av, |  | Saline, App/Av, Area 32 | Saline, App/Av, Area 32 | 5HT1a, BL reward, Area 25 |
| 16 | Musbac, BL reward Area 32 |  | 5HT1a, App/Av, Area 25 | LY/CGP, App/Av, Area 32 | 5HT1a, App/Av, Area 25 |
| 17 | LY/CGP, App/Av, Area 32 |  | Musbac, BL reward, Area 32 | LY/CGP, BL reward, Area 32 |  |
| 18 | LY/CGP, BL reward, Area 32 |  | Musbac, App/Av, Area 32 | Saline, BL reward, aHipp |  |
| 19 | 5HT1a antag, App/Av, Area 25 |  | Musbac Area 32, LY/CGP aHipp, App/Av, | Saline, App/Av  Area 25 (repeat) |  |
| 20 | Saline, BL reward, aHipp |  | 5HT1a, BL reward, Area 25 | 5HT1a antag, App/Av, Area 25 |  |
| 21 | 5HT1a, BL reward, Area 25 |  | LY/CGP, BL reward, aHipp | Musbac Area 32 & LY/CGP aHipp, App/Av, |  |
| 22 |  |  | Musbac, BL reward, aHipp | 5HT1a antag, BL reward, Area 25 |  |
| 23 |  |  | Saline, BL reward, aHipp | Musbac Area 32 & LY/CGP aHipp, App/Av (repeat) |  |
| 24 |  |  |  | LY/CGP, BL reward, aHipp |  |
| 25 |  |  |  | Musbac, BL reward, aHipp |  |

**Figure S1. Individual baseline performances across the whole experiment.**

All marmosets show relatively stable performance across the whole study when looking at measures of total responses (A), the average number of responses required to earn each reward (B), and the baseline spatial bias (responses to one side divided by the responses to the other side; C). To avoid confounds of drug infusions, the data presented portrays the performance of each animal on the day before each infusion.


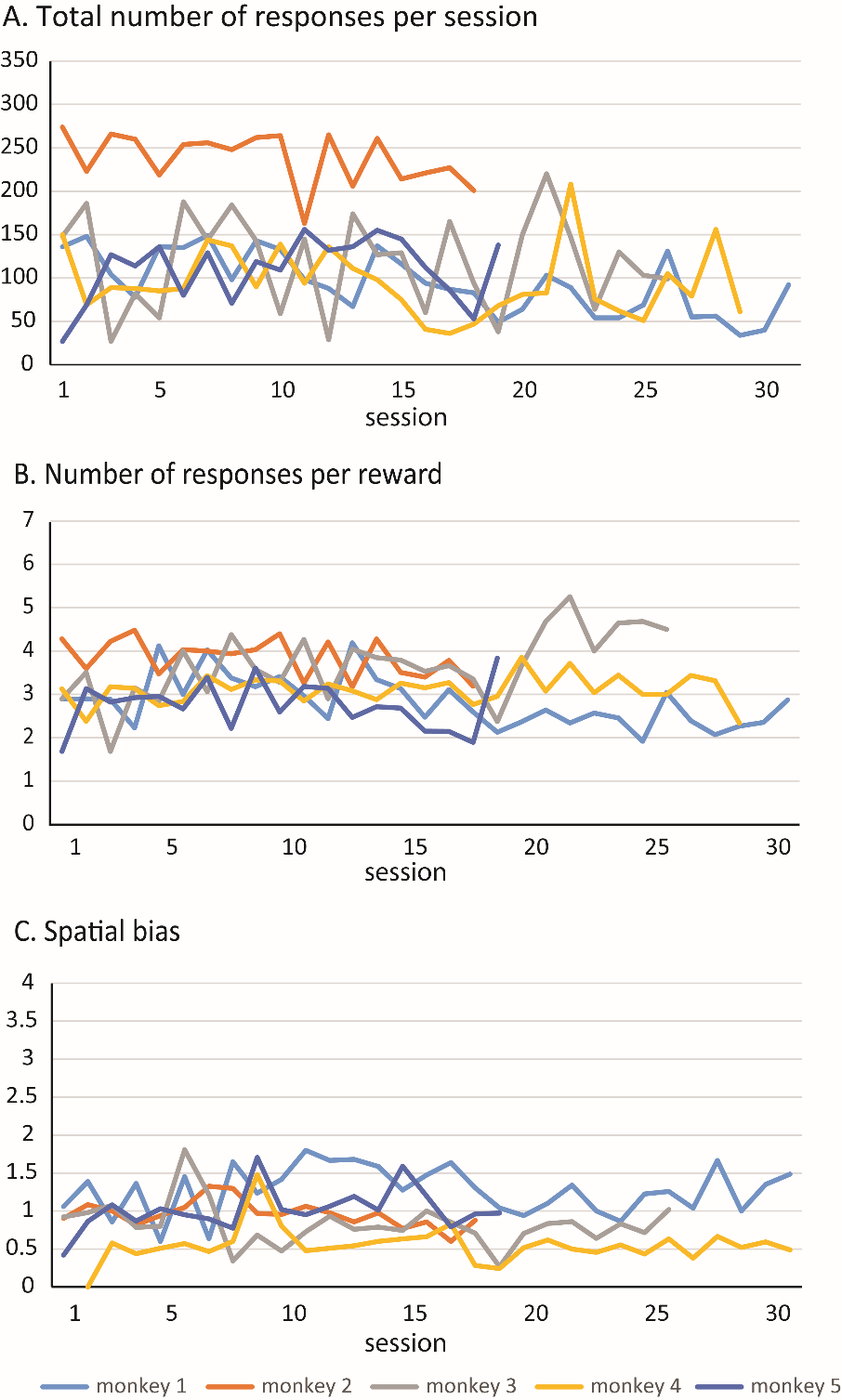


**The number of rewards obtained is not altered by reductions in responding, but does rise when responding increases.**

In contrast to the pronounced effects of pharmacological manipulations of the aHipp and areas 25 and 32 on the number of responses made, these findings did not translate into equivalent alterations in the number of rewards obtained. However, this is predicted by the VI schedules as only occasional responding to a particular side is required to obtain all the rewards associated with that side. As the maximum reward interval is 20 seconds, responding once every 20 seconds to a particular side for a duration of 10 mins would be expected to produce 30 responses and maximum of 30 rewards. However after saline infusions, the monkeys respond more than this and make approximately one response every 3 seconds across both sides (0.35 ± 0.068 responses per second; calculated from the average response rate of all animals after saline infusions averaged across all three areas). Thus across 10 mins, they would be expected to respond 200 times (not excluding reward consumption time), and assuming relatively equal distribution of these responses to both sides, would therefore make approximately 100 responses per side. However because the VI schedule limits the number of rewards available regardless of the number of responses, this 100 responses would still only result in 30 rewards. Consequently a drop in responding from 100 to below 30 would be required before any reduction in the number of rewards is seen, and a drop of this magnitude is larger than the usual alterations in response number seen (see Fig S1A). Thus repeated measures ANOVA of the pharmacological manipulation of the aHipp revealed no condition x drug interaction (F_2,18.05_, = 0.417, P = 0.66), and similar analysis of areas 25 and 32 revealed no condition x drug x area interaction (F_2, 37.6_, = 0.11, P = 89). Similarly, no changes in reward number were seen after 5-HT1a administration in area 25 (F_1,12_ = 2.42, P = 0.145) or after aHipp LY/CGP + musbac 32 (F_2,10_, = 0.01, P = 0.98) or aHipp LY/CGP + 25 WAY (F_2,6.49_ = 3.69, P = 0.085). See Figure S2A.

Although drops in responding are unlikely to lead to decreases in the number of rewards, it is possible for increases in responding to increase the number of rewards. Optimal responding on a VI schedule involves the animals making a response immediately after each delay has timed out. This serves to minimise the gaps between successive reward intervals and maximises the number of rewards that can be obtained. Clearly, the VI schedule means the animals cannot deliberately do this, as they never know when the end of the delay in approaching. But, if an animal who is responding sub-optimally increases their rate of responding, the increased number of responses will mean that they are more likely to respond closer to the end of each delay, and should therefore obtain more reward intervals and their associated rewards. In the current data set, the monkeys only received an average of 22.4 (± 0.79) rewards on their preferred side (calculated from the average number of rewards obtained on the preferred side of all animals after saline infusions from all three areas in the baseline reward condition) indicating that they were responding sub-optimally. An increase in the number of responses was also seen after inactivation of area 25. Consequently, ANOVA of reward number revealed a main effect of area 25 inactivation on the effects of aHipp LY/CGP, with area 25 inactivation increasing the number of rewards obtained compared to aHipp LY/CGP alone (main effect of manipulation, F_2,5.7_ = 27.315, P = 0.001; aHipp LY/CGP + musbac 25 vs aHipp LY/CGP; t_3_ = 3.332, P = 0.045; aHipp LY/CGP + musbac 25 vs musbac 25; t_3_ = 0.902, P = 0.434; aHipp LY/CGP vs musbac 25; t_3_ = 5.572, P = 0.011). Similarly, despite the lack of effect of 25/32 manipulations on reward number, targeted investigation reveals that inactivation of area 25 also increases reward number compared to saline in both the baseline reward and approach/avoidance conditions, although these increases were not quite significant (baseline reward; t_4_ = 2.746, P = 0.052; approach/avoidance; t_4_ = 2.643, P = 0.057).

Analysis of the number of rewards obtained on the preferred and non-preferred sides also revealed no main effects of condition x drug x side preference after pharmacological manipulation in the aHipp (F_2,40.01_, = 0.088, P = 0.91), no effects of area x condition x drug x side preference after manipulation of areas 25 and 32 (F_2,67.8_, = 0.37, P = 0.69), and no effects of the combined manipulations (all Fs<1, NS. highest F =0.29). See Figure S2B.

**Figure S2 legend**

Independent pharmacological manipulation of the aHipp, area 25 and area 32 produced differential effects on how many rewards were obtained in the baseline reward (blue drop only) and approach/avoidance (blue drop and bell) conditions. Figures show the drug-induced changes in the absolute reward number and the relative number of rewards obtained on the preferred and non-preferred sides compared to saline controls. Data are represented as mean ± SEM. † trend effect of manipulation. *P< 0.05.

Overall analysis of the manipulations within each area revealed no alterations in reward number in the aHipp (**Ai**), areas 25 (**Aii**) and 32 (**Aiii**). However targeted analysis revealed that an increased number of rewards after area 25 inactivation that trended towards significance. Similarly, the combined aHIpp LY/CGP + musbac 25 increased the number of rewards obtained, although aHipp LY/CGP + 25 WAY and aHipp LY/CGP + musbac 32 were unaffected (**Aiv**).

The number of rewards obtained on the preferred/punished and non-preferred sides was unaltered (**B**).

Musbac, muscimol/baclofen-mediated inactivation; LY/CGP, LY341495/CGP52432-mediated presynaptic glutamatergic disinhibition; WAY, WAY100135-mediated 5-HT1a antagonism.

Figure S2


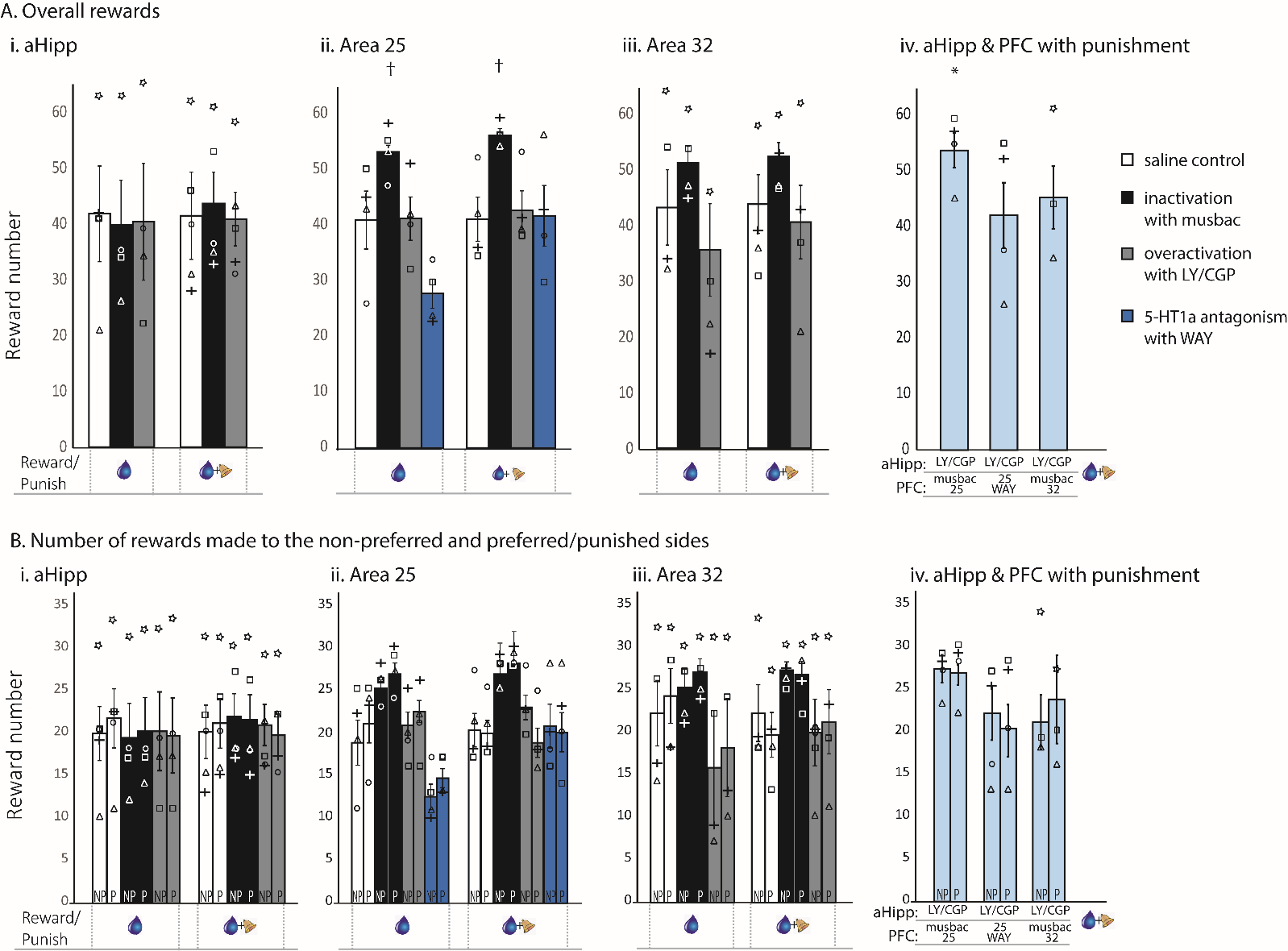

Supplement: bhz015_Clarke_Supplementary [file bhz015_clarke_supplementary.docx]
